# In Vitro Toxicity Study of a Porous Iron(III) Metal‒Organic Framework

**DOI:** 10.3390/molecules24071211

**Published:** 2019-03-28

**Authors:** Gongsen Chen, Xin Leng, Juyuan Luo, Longtai You, Changhai Qu, Xiaoxv Dong, Hongliang Huang, Xingbin Yin, Jian Ni

**Affiliations:** 1 School of Chinese Materia Medica, Beijing University of Chinese Medicine, Beijing 102488, China; gongsenchen@163.com (G.C.); 20160931927@bucm.edu.cn (X.L.); luojy0329@163.com (J.L.); ylt_svip@163.com (L.Y.); quchanghai@bucm.edu.cn (C.Q.); dxiaoxv@163.com (X.D.); 2 Beijing Research Institute of Chinese Medicine, Beijing University of Chinese Medicine, Beijing 100029, China; 3 State Key Laboratory of Separation Membranes and Membrane Processes, Tianjin Polytechnic University, Tianjin 300387, China; 4 School of Chemistry and Chemical Engineering, Tianjin Polytechnic University, Tianjin 300387, China

**Keywords:** MIL-100(Fe), in vitro toxicity, HL-7702 cells, HepG2 cells

## Abstract

A MIL series metal‒organic framework (MOF), MIL-100(Fe), was successfully synthesized at the nanoscale and fully characterized by TEM, TGA, XRD, FTIR, DLS, and BET. A toxicological assessment was performed using two different cell lines: human normal liver cells (HL-7702) and hepatocellular carcinoma (HepG2). In vitro cytotoxicity of MIL-100(Fe) was evaluated by the MTT assay, LDH releasing rate assay, DAPI staining, and annexin V/PI double staining assay. The safe dose of MIL-100(Fe) was 80 μg/mL. It exhibited good biocompatibility, low cytotoxicity, and high cell survival rate (HL-7702 cells’ viability >85.97%, HepG2 cells’ viability >91.20%). Therefore, MIL-100(Fe) has a potential application as a drug carrier.

## 1. Introduction

Metal‒organic frameworks (MOFs) are the latest class of crystalline hybrid materials developed over the last 20 years [1]. MOFs are built from virtually infinite combinations of metal subunits and organic ligands (aromatic acids or bases) through coordination bonds, and are also named porous coordination polymers (PCPs) [2,3,4]. MOFs are rich in composition, crystal structure, and crystal shape, and have irreplaceable advantages compared with other types of materials [5]. The high versatility of MOFs is shown by their various applications over the past 10 years, including catalysis [6], sensing [7], gas storage [8], bio-imaging [9], and magnetism [10]. Because of their large specific surface area, connectivity polyhedron, high pore volume, high stability, non-toxicity, biocompatibility, and small size, MOFs have become the most promising nanocarriers in the biomedical field [11,12,13,14]. Therefore, MOFs can be used in a variety of designs, such as imaging agents for early and minimally invasive diagnostics [15], specific cell and tissue (more commonly tumor tissue) targeting [16], high drug loading [17], increased local drug concentration [18], and developing drug delivery preparations [19]. As described above, nanomedical MOFs are considered promising candidates for developing more effective therapies and reducing side effects.

However, the increasing potential uses of MOFs may pose health risks to the patient. Therefore, this has led to controversies about whether MOFs are biocompatible in biomedical applications. To date, the safety assessment of MOFs has been largely limited by the metals and linkers. Preliminary studies showed that, owing to differences in chemical formulations, the toxicity of Zn, Zr, Mg, and Fe, as assessed by LD50, rose from several μg/kg to more than 1 g/kg [20]. The organic ligands, such as the commonly used polycarboxylic acids, are less toxic and easily removed under physiological conditions because of their high polarity [21,22]. The diversity of metal clusters and organic ligands, as well as the functional modification inside and outside the surface, represents further advantages of MOFs, but can also change the chemical and physical properties of materials [23,24]. The range of cytotoxicity, hydrophobic/hydrophilic balance, host‒guest interaction, body distribution, biodegradability, tissue accumulation, and excretion determines their toxicity and whether they could be used for medical applications. Therefore, before the practical application of any new MOFs, a detailed study of their toxicity and biosafety is urgently needed. In particular, it is required that MOFs have minimal interference with the main effector cells [25], so the study of their possible toxic effects is very important.

**Figure 1:**
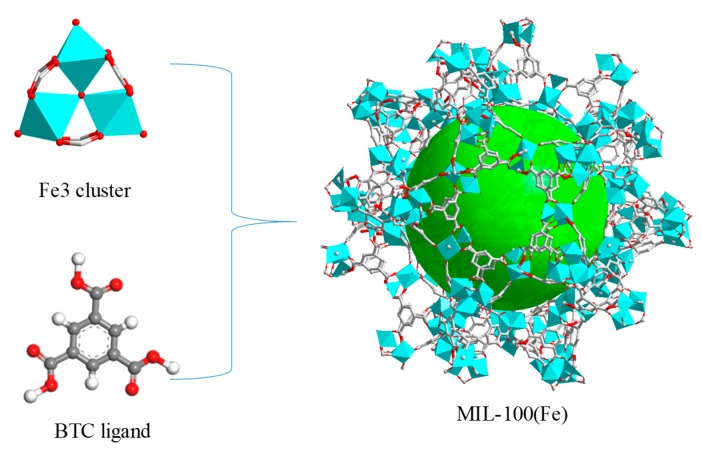
Schematic illustration of the construction of MIL-100(Fe).

Up to this point, among the thousands of MOFs reported, the most commonly used biomedical materials are MIL (Material Institute Lavoisier) series [26]. MIL-100(Fe) is synthesized from FeCl_3_.6H_2_O and H_3_BTC under hydrothermal conditions. The molecular formula is Fe_3_(H2O)_2_O-BTC and the nanoporous structure is shown in Figure 1. There are two kinds of mesoporous cages in the skeletal structure with pore diameters of 2.5 nm and 2.9 nm [27,28,29]. MIL-100(Fe) has been widely studied as an anticancer drug carrier owing to its high specific surface area (1700 BET), high porosity, large drug-loading capacity, stability in a biological medium, and eco-friendly nature [30,31]. For instance, MIL-100(Fe) has a surface functionalization of maghemite nanoparticles, encapsulated doxorubicin with 14 wt % loading, and progressive delivery of drug (PBS, 37 °C) exceeding 25 days [32]. Doxycycline monohydrate and tetracycline hydrochloride, loaded separately on nano-MIL-100, were able to give a sustainable release at SGF and PBS [33]. The effect of pH and temperature on the adsorption of cephalexin and release behavior of MIL-100(Fe) was studied [34]. Docetaxel was encapsulated in MIL-100(Fe) with a drug payload up to 57.2 wt %; the release rate of the drug after 96 h was measured in neutral (pH 7.4, 5.08%) and acidic (pH 5.5, 15.53%) PBS that included polysorbate 80 (1% *v*/*v*) [35]. To date MOFs have, surprisingly, not been comprehensively analyzed for their adverse effects on hepatic cells, but have only been preliminarily studied for their in vitro toxicity in cancer cells (see Table 1). As is well known, the liver is a detoxifying organ, and nanomaterials tend to accumulate in the liver tissue after entering the body [36]. Therefore, it is important to determine whether they cause hepatotoxicity.

The toxic effect of MIL-100(Fe) on different types of cells varies greatly, so one must ensure the biocompatibility of MIL-100(Fe) to target cells and that its intrinsic toxicity does not exceed the benefits of reducing the toxicity of drugs or treatments. The in vitro toxicity test is fast, effective, and has a low cost. It provides information about the possible interaction between cells and nanoparticles to evaluate the potential hazards of nanomaterials. Therefore, these assays are appropriate for HTS (high-throughput screening) of most new medical nanomaterials and are useful in establishing cytotoxicity levels [39]. In accordance with the above statements, we synthesized MIL-100(Fe) by the solventthermal method and focused on the influence of MIL-100(Fe) on two types of hepatocytes to analyze its cytotoxicity in vitro. The synthesized MIL-100(Fe) was analyzed by TEM, TGA, XRD, FTIR, DLS, and BET. The in vitro cytotoxicity of MIL-100(Fe) in HL-7702 and HepG2 cells was evaluated by the MTT assay, the LDH releasing rate assay, DAPI staining, and annexin V/PI double staining assay. In conclusion, our study evaluates the in vitro safety of MIL-100(Fe) and provides a reference for medical applications.

## 2. Results

### 2.1. Preparation and Characterization of MIL-100(Fe)

**Figure 2:**
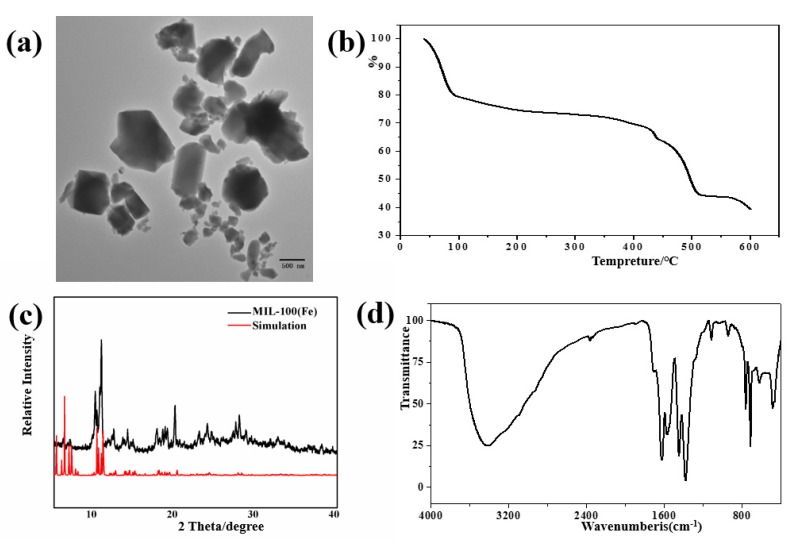
Characteristics of MIL-100(Fe): (**a**) TEM, (**b**) TG, (**c**) XRD, (**d**) FTIR.

According to previous studies, MIL-100(Fe) was synthesized by the hydro-thermal method. The structure of MIL-100(Fe) was analyzed by TEM, TGA, XRD, FTIR, DLS, and BET. The TEM image of the synthesized materials, shown in Figure 2a, shows irregular polyhedron-shaped nanoparticles for MIL-100(Fe) of 200–500 nm diameter, similar to the reported results [25].

As shown in Figure 2b, in the temperature range of 40–600 °C, the first weight loss occurs between 80 and 150 °C (about 23 wt %) due to the volatilization of free water in the material. In the range of 150–350 °C, there is a slow weight loss, which is due to the release of some anionic small molecules used to balance the skeleton charge and bound water interrelated with the iron metal trimers. The third weight loss occurs at 350–500 °C, with the largest reduction in weight (about 30 wt %) mainly due to the collapse of the sample frame structure and the decomposition of organic connectors [40]. The above thermogravimetric characteristics match MIL-100 (Fe).

The XRD patterns of the synthesized materials are presented in Figure 2c. Compared with the MIL-100(Fe) simulated pattern, the diffraction peaks of the experimental group match well, which indicates that the synthesis of MIL-100(Fe) crystal is successful.

According to Figure 2d, peaks near 1624 and 1381 cm^−1^ belong to the stretching vibrations of C=O. In addition, bending vibration of aromatics is observed at 760 and 712 cm^−1^, The wide absorption near 3500 cm^−1^ is due to the O–H stretching vibrations of H_2_O molecules. All the above are characteristic peaks of MIL-100(Fe) [38].

**Figure 3:**
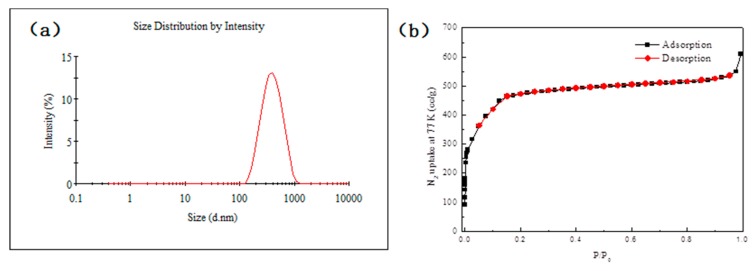
(**a**) Size distribution of MIL-100(Fe); (**b**) BET isotherm of MIL-100(Fe) at 77 K.

The particle size distribution of MIL-100(Fe) was measured by a Malvern Zetasizer Nano ZS 90 (Malvern, UK). The particle size analysis (Figure 3a) shows an average particle size of 345.3 nm (Pdi: 0.181; Intercept: 0.956). The porosity of MIL-100(Fe) was characterized by N_2_ adsorption–desorption isotherms at 77 K, as shown in Figure 3b. The Brunauer–Emmett–Teller (BET) specific surface area is 1750 m^2^/g and the pore volume is 0.83 cc/g, large enough for efficient mass transfer of drug loading and releasing.

### 2.2. Effect of MIL-100(Fe) on HL-7702 Cells

**Figure 4:**
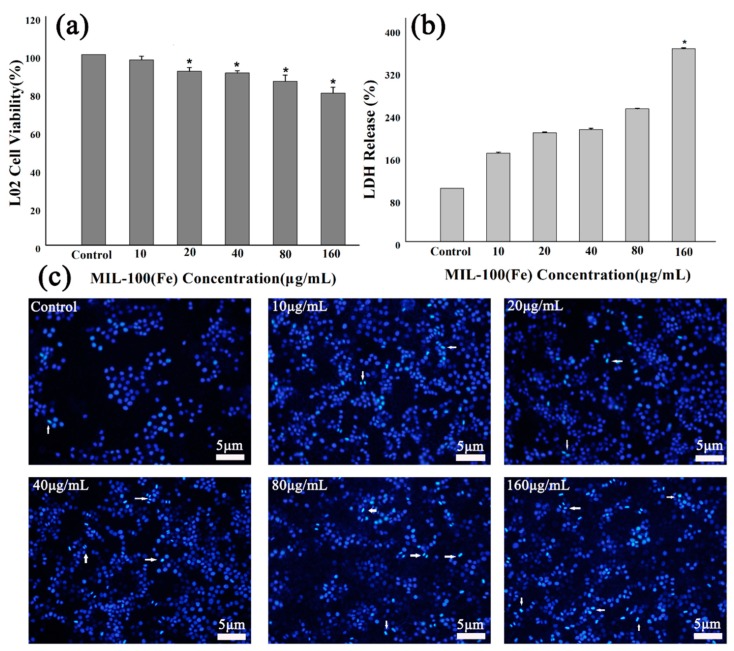
Effects of MIL-100(Fe) on HL-7702 cells’ viability and morphology: (**a**) MTT assay data, presented as mean ± SD of viability% of three independent experiments; (**b**) the effect of MIL-100(Fe) on LDH release of HL-7702 cells; (**c**) an evaluation of HL-7702 cells nuclear morphology by DAPI staining. (* *p* < 0.05 vs. Control.)

The MTT assays were carried out on HL-7702 cells. The cells were treated with different concentrations of MIL-100(Fe) and the toxicity was determined (Figure 4a). The results showed that MIL-100(Fe) particles with a concentration of less than 80 μg/mL were nontoxic (cell viability >85%). Moreover, the amount of LDH was measured in the medium, which was regarded as an important indicator of cell membrane integrity, as illustrated in Figure 4b. MIL-100(Fe) of high concentrations (160 μg/mL) was toxic to HL-7702 cells and would destroy the cell membrane, leading to significant LDH release. Fortunately, cells treated with 160 μg/mL MIL-100(Fe) for 48 h retain a cell survival rate of 79.81 ± 3.01%, and IC50 is up to 1.37 mg/mL. DAPI staining for HL-7702 cells did reveal a high concentration of MIL-100(Fe)-induced morphological changes associated with apoptosis, as demonstrated in Figure 4c.

**Figure 5:**
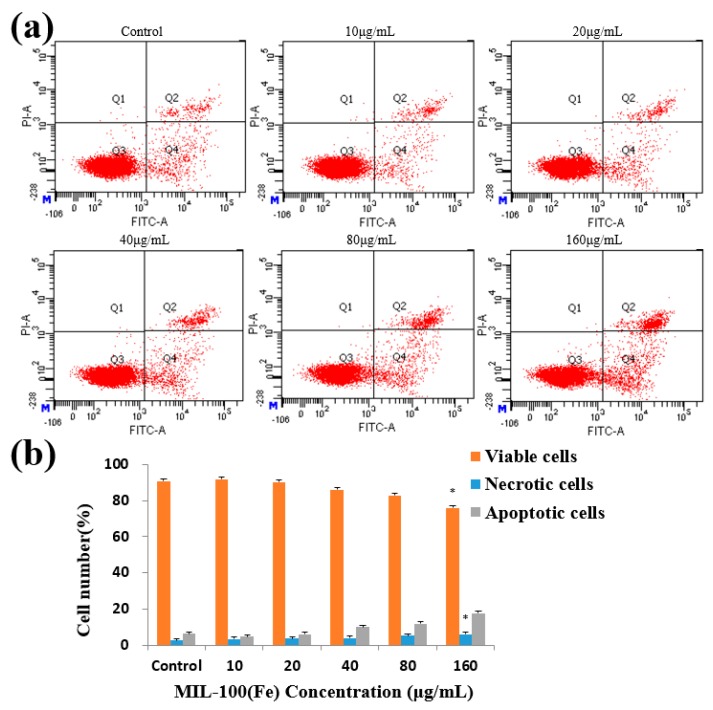
Effects of MIL-100(Fe) on apoptosis in HL-7702 cells. (**a**) Flow cytometry detection of apoptosis with FITC-annexin V/PI double staining. (**b**) The percentages of viable, necrotic, and apoptotic HL-7702 cells after incubation with different concentrations of MIL-100(Fe) for 48 h. The data are expressed as means ± S.D. from three independent experiments. (* *p* < 0.05 vs. Control.)

After the treatment of HL-7702 cells with MIL-100(Fe) for 48 h, the higher dose of MIL-100(Fe) (160 μg/mL) had a significant effect on the apoptosis of HL-7702 cells (Figure 5). The percentage of necrotic cells only reached 6.07%. The results indicated that MIL-100(Fe) particles had good biocompatibility with HL-7702 cells.

### 2.3. Effect of MIL-100(Fe) on HepG2 Cells

**Figure 6:**
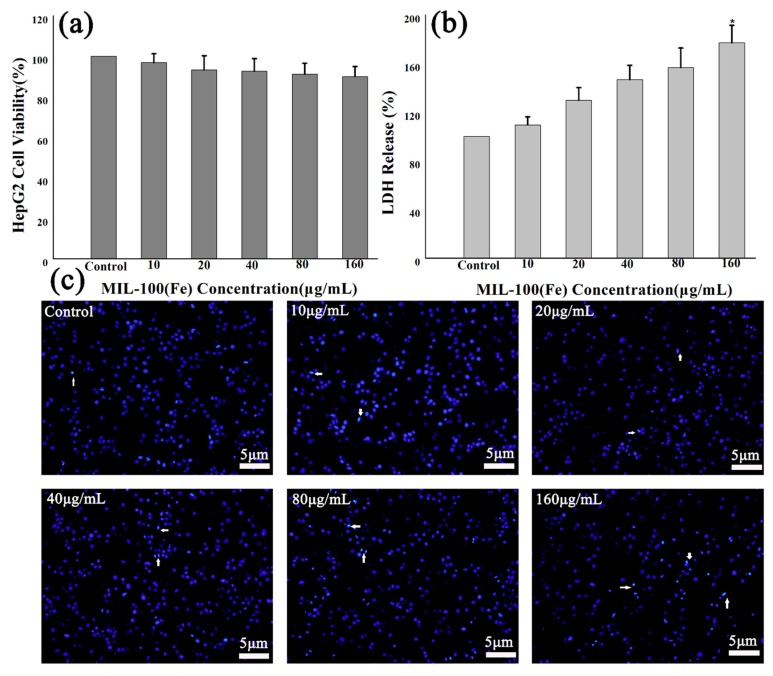
Effects of MIL-100(Fe) on HepG2 cells’ viability, membrane, and morphology: (**a**) In vitro cell viabilities of HepG2 cells after being incubated for 48 h with MIL-100(Fe); (**b**) LDH release of HepG2 cells incubated for 48 h with MIL-100(Fe); (**c**) nuclear morphology images of HepG2 cells after 48 h exposure to MIL-100(Fe). (* *p* < 0.05 vs. Control.)

The MTT toxicity test was performed by treating HepG2 cells with different concentrations of MIL-100(Fe). As depicted in Figure 6a, MIL-100(Fe) shows no cytotoxic effect on the cells. It reveals 89.98 ± 4.96% cell viability at the high concentration (160 μg/mL). Remarkably, the LDH release assay showed that HepG2 cells had good tolerance to MIL-100(Fe), even at higher doses (Figure 6b). At a MIL-100(Fe) concentration of 160 μg/mL, LDH release was significantly increased, but there was no significant decrease in cellular metabolic activity, indicating that they were well tolerated. DAPI staining of HepG2 cells indicated that high concentrations of MIL-100(Fe) did induce nucleus morphological changes, as shown in Figure 6c.

**Figure 7:**
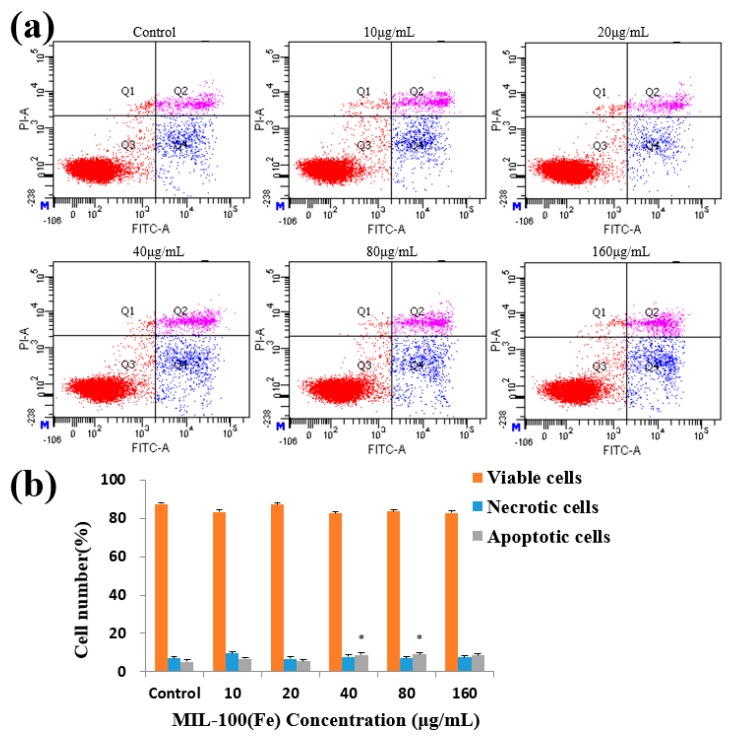
Effects of MIL-100(Fe) on apoptosis in HepG2 cells. (**a**) Flow cytometry detection of apoptosis with FITC-annexin V/PI double staining. (**b**) The percentages of viable, necrosis and apoptotic HepG2 cells after incubation with different concentrations of MIL-100(Fe) for 48 h. The data are expressed as means ± S.D. from three independent experiments. (* *p* < 0.05 vs. Control.)

After a 48-h treatment of HepG2 cells with MIL-100(Fe), there was no significant change in the percentage change of viable cells and early apoptotic cells. The late apoptotic cells were significantly affected at concentrations of 40–80 μg/mL, but the overall difference was small (Figure 7). MIL-100(Fe) had little effect on cells’ morphological changes, indicating the good biocompatibility of MIL-100(Fe) with HepG2 cells.

## 3. Discussion

This study was designed to assess the potential toxicity of MIL-100(Fe) and explore its safe dose. To date, few studies have used normal cells to study the toxic effects of MIL-100(Fe), so there is currently no comparison of the effects of MIL-100(Fe) between normal hepatocytes and liver cancer cells. Recently, Wang et al. [41] demonstrated a low toxicity of zinc-based MOFs by MTT experiments, and also tested drug loading and evaluated the efficacy. Another report by Sun et al. [42] used a novel Gd(III) MOF-loaded anticancer drug, and, using MTT, evaluated its toxicity to both cell lines and its anticancer activity after drug delivery. The cytotoxicity of these materials depends not only on the composition of the MOFs, but also on the choice of cell model. In other words, different MOFs have different toxicities to different cell lines. The current study aimed to determine the toxicity of MIL-100(Fe) on normal human liver cells and liver cancer cells and compare them.

We recently reported the in vitro hepatotoxicity of MIL-53(Fe), loaded the antitumor drug oridonin, and conducted in vitro sustained release and pharmacodynamic studies [43]. Although in vitro cell models are not a substitute for animal experiments, they can serve as a basis for further evaluation of the potential toxicity.

Our results showed that both cell lines, which were treated with the same concentration gradient of MIL-100(Fe) for 48 h, showed a dose-dependent increase in LDH leakage and a decrease in cell viability. Compared with HepG2 cells, HL-7702 cells were significantly affected by the dose change of MIL-100(Fe), which showed strong cytotoxicity (cell viability <85%) at a high dose (160 μg/mL). With the same treatment, HepG2 cells had less damage. Both cell types showed obvious membrane damage (LDH leakage) at the highest concentration. LDH enzymes are highly sensitive indicators of cellular metabolic status, aerobic or anaerobic directions of glycolysis, activation status, and malignant transformation [44]. It is unclear whether the rapid sedimentation of MIL-100(Fe) particles in the medium has an effect on the state of the cells. Normal hepatocytes and liver cancer cells have different tolerances to MIL-100(Fe) particles, and it is unclear whether this is related to cell metabolism or cell membrane changes after carcinogenesis [45]. Overall, based on MTT and LDH data, MIL-100(Fe) showed low toxicity to HepG2 and HL-7702 cells at a dose of 80 μg/mL.

Additional studies were performed using DAPI staining to observe changes in the nuclear morphology of the two cell lines after MIL-100(Fe) treatment. In a study on the role of polyadenylation in apoptosis and drug resistance of K562 cells, Lallas used DAPI staining to detect apoptotic cell nuclear division [46]. DAPI (4′,6-diamidino-2-phenylindole) is a fluorescent dye that binds strongly to DNA and can be utilized for staining living and fixed cells [47]. Our results show that the nuclear morphological changes of the two cell lines gradually became obvious with an increased dose, indicating that a high concentration of MIL-100(Fe) induced morphological changes related to apoptosis. annexin V combined with PI can distinguish early apoptotic cells from late apoptotic and dead cells, and is the ideal quantitative detection method for apoptosis at present [48]. Han et al. [49] used the MTT assay to detect the biocompatibility of the *Zr*-based MOF, UiO-66, against SMMC-7721 and HeLa cells, and evaluated the apoptosis-inducing effect of AgNPs@UiO-66 by annexin V/PI staining. To more clearly elucidate the effects of apoptosis, we performed annexin V/PI staining. Our results indicate that the percentage of apoptotic normal hepatocytes increases in a dose-dependent manner as the concentration of MIL-100(Fe) increases, with significant differences at the highest dose. It has been demonstrated that MIL-100(Fe) particles at a dose of 80 μg/mL had little effect on the degree of apoptosis in the two cell models. There are several problems with determining how high concentrations of MIL-100(Fe) particles induce toxicity. For example, we need to address the questions of whether the particles enter the cell or adhere to the cell membrane, and whether the toxicity of materials with different shapes and sizes is the same. MIL-100(Fe) is one of the most studied MOFs today, and its toxicity and safe dose are currently unclear. It is therefore necessary to study their toxicology, and this report provides some preliminary information in this regard.

Due to the complexity of toxicity, the next step is to study whether the toxicity of MIL-100(Fe) is related to intracellular pathways such as autophagy, mitochondrial membrane potential change, lysosomal membrane permeability, and endoplasmic reticulum stress, so as to more comprehensively evaluate the toxicity mechanism of MIL-100(Fe).

## 4. Conclusions

In conclusion, high concentrations of MIL-100(Fe) particles resulted in decreased viability of HepG2 and HL-7702 cells, LDH leakage, and induction of apoptosis. The safe dose of MIL-100(Fe) was determined to be 80 μg/mL by the MTT assay, the LDH release assay, the DAPI staining assay, and the annexin V/PI double staining assay. MIL-100(Fe) particles had excellent biocompatibility with HL-7702 and HepG2 cells, low cytotoxicity, and allowed a high cell survival rate. Therefore, the metal organic framework material MIL-100(Fe) has a potential application as a drug carrier.

## 5. Experimental Section

### 5.1. Materials

All general reagents and solvents (AR grade) were commercially available. Ethanol, nitric acid (HNO3, 65%~68%), and hydrofluoric acid (HF ≥ 40%) were purchased from Beijing Chemical Works (Beijing, China). 1,3,5-benzenetricarboxylic acid (H3BTC, 99%) was supplied by TCI (Shanghai, China). Iron powder (Fe ≥ 99.5%) was obtained from Aladdin Chemistry Co. Ltd. (Aladdin, Shanghai, China). DMEM, FBS, penicillin-streptomycin solution and 0.25% trypsin were purchased from Corning (Corning, NY, USA). PBS was obtained from Solarbio (Beijing, China). MTT was acquired from Biotopped (Beijing, China). The LDH Assay Kit, DAPI Assay Kit, and annexin V-FITC Apoptosis Assay Kit were purchased from Beyotime (Nanjing, China).

### 5.2. Synthesis and Characterization of MIL-100(Fe)

MIL-100(Fe) was synthesized and activated according to previous studies [50,51]. Typically, Fe powder (0.82 g), H3BTC (2.06 g), HF (40%, 0.6 mL), HNO3 (65%, 1.14 mL), and deionized water (80 mL) were mixed in a Teflon liner (Yushen, Shanghai, China), which was placed in an oven, and progressively heated to 150 °C within 8 h. The temperature was maintained at 150 °C for 96 h and then the oven was cooled down to room temperature for 24 h. The resulting orange powder was collected after filtration and then washed with water. To remove the residual ligand and metal ions from the pore of the MOF, the sample was dispersed in hot water (80 °C) for 8 h (about 1 g sample in 300 mL water) and then in hot ethanol for 8 h. Finally, the solid was dried overnight in a vacuum at 120 °C.

### 5.3. Cell Culture

HL-7702 cells and HepG2 cells were purchased from Guangzhou Jeniobio Biotechnology (Beijing, China). The cells were cultured in a high-glucose DMEM medium supplemented with 10% (*v*/*v*) FBS and 1% (*v*/*v*) penicillin (100 µg/mL)‒streptomycin (100 µg/mL) at 37 °C in a humidified atmosphere containing 5% CO_2_. Cells were passaged at 1:3 after 3–4 days. MIL-100(Fe) was dissolved in DMEM to a stock concentration of 2 mg/mL, and after being dispersed by ultrasonic and ultraviolet disinfection was stored at 4 °C. The cells were treated with different doses of MIL-100(Fe) after dilution in the basal medium.

### 5.4. Cell Viability Assay

The effect of MIL-100(Fe) on HL-7702 and HepG2 cells was assessed by the MTT assay. Cells were seeded into 96-well plates at a density of 3.0 × 10^3^ cells/well overnight. The cells were treated with 0, 10, 20, 40, 80, and 160 µg/mL MIL-100(Fe) for 48 h. Fresh basal medium was used as the untreated control. Then, 20 µL MTT working solution (5 mg/mL) was added. After incubation for 4 h at 37 °C, culture supernatant was removed from all the wells, and the formazan crystals were dissolved in 150 µL DMSO. The supernatant (100 µL) was transferred into new 96-well plates after centrifugation (1000 g, 5 min). A microplate reader (Multiskan GO, Thermo, Waltham, MA, USA) was used to measure the absorbance at 570 nm. All the experimental results were carried out three times.

### 5.5. Cell Membrane Damage Determination

LDH is an enzyme that predominantly exists in the cytoplasm, and is released into the extracellular medium when the cell membrane is damaged. Therefore, the cell membrane damage was determined by LDH leakage (LDH%). For the measurement of LDH leakage, the LH-7702 and HepG2 cells were seeded into 96-well plates at a density of 5.0 × 10^3^ cells/well overnight and then treated with various concentrations of MIL-100(Fe) for 48 h. The supernatant was collected and LDH activity was measured with a commercial kit following the manufacturer’s instructions. The experiments were conducted in triplicate.

### 5.6. DAPI Fluorescence Staining

Morphological changes of chromatin condensation and nuclear fragmentation could be observed by DAPI staining. HL-7702 and HepG2 cells at a density of 3.5 × 10^5^ cells/well were plated in six-well plates and treated with 0, 10, 20, 40, 80, and 160 µg/mL MIL-100(Fe) for 48 h. The culture supernatant was discarded and the cells were fixed with 4% paraformaldehyde (500 mL) for 10 min at room temperature. Then the fixed cells were washed two times with PBS and stained with DAPI solution (2.5 µg/mL, 0.8 mL) for 10 min. After being washed twice with PBS, the morphological changes were photographed using an inverted Olympus IX71 fluorescence microscope (Tokyo, Japan) at 200×.

### 5.7. Apoptosis Analysis

Apoptosis usually refers to a type of programmed cell death in the process of development or under the action of various factors, through the regulation of genes and their products in cells, and can be detected by an annexin V-FITC detection kit [52]. HL-7702 and HepG2 cells were plated in a six-well plate (3.5 × 10^5^ cells/well) and incubated with gradient concentrations (0, 10, 20, 40, 80, and 160 µg/mL) of MIL-100(Fe) for 48 h at 37 °C. The cells were gathered and washed with PBS. Then, the cells were resuspended in 295 µL binding buffer and incubated with 5 µL annexin V-FITC and 10 µL PI at room temperature, hatching 20 min and blocking the light ray. All the samples were analyzed by flow cytometry (BD FACS Canto II, Franklin Lakes, NJ, USA) immediately.

### 5.8. Statistical Analysis

Each experiment was repeated three times, and the results were expressed as mean ± SD. By using SAS 9.4 software (Beijing, China), the statistical analysis was carried out with the further statistical methods of single factor analysis of variance, multivariate comparison, and a non-parametric test. A value of *p* < 0.05 was regarded as significant.

## Figures and Tables

**Table 1:** The Effect of MIL-100 (Fe) on Various Cells, 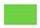: biocompatible; 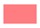: adverse effects.

MOFs and MOFs-Objects	Cells	Dose (μg/mL)	Cell Viability	Toxic Grade	Reference
MIL-100(Fe)	PC3	100 (24 h)	75%	low	Saad et al. [32]
MIL/USPIO-cit(10)	PC3	100 (24 h)	75%	low	Saad et al. [32]
MIL-100(Fe)	HUVEC	200 (24 h)	100%	none	Stefan et al. [37]
	HMEC	200 (72 h)	85%	low	Stefan et al. [37]
	MLE12	200 (24 h)	10%	high	Stefan et al. [37]
	MH-S	200 (24 h)	10%	high	Stefan et al. [37]
	Gingival Fibroblasts	200 (24 h)	100%	none	Stefan et al. [37]
	Human Schwann	200(24 h)	60%	medium	Stefan et al. [37]
MIL-100(Fe)@DOPC	HUVEC	200 (24 h)	100%	none	Stefan et al. [37]
	HMEC	200 (72 h)	60%	medium	Stefan et al. [37]
	MLE12	200 (24 h)	40%	high	Stefan et al. [37]
	MH-S	200 (24 h)	20%	high	Stefan et al. [37]
nanoMIL-100(Fe)	MCF-7	100 (72 h)	94%	none	Mahsa et al. [35]
MIL-100(Fe)	CCRF-CEM	5000(48 h)	>85%	low	Patricia et al. [38]
	RPMI-8226	5000(48 h)	>85%	low	Patricia et al. [38]
	J744	5000(48 h)	>85%	low	Patricia et al. [38]

## Abbreviations

MOFs	Metal‒organic frameworks
PCP	Porous coordination polymer
LD_50_	Lethal dose 50%
MIL	Material Institut Lavoisier
H_3_BTC	1,3,5-Benzenetricarboxylic acid
PBS	Phosphate-buffered saline
SGF	Simulated gastric fluid
PC3	Human prostate cancer cell line
HUVEC	Human umbilical vein endothelial cells
HMEC	Human microvascular endothelial cells
MLE12	Murine alveolar epithelial cells
MH-S	Mouse alveolar macrophage cell line
MCF-7	Human breast adenocarcinoma cell line
CCRF-CEM	Human leukemia
RPMI-8226	Human multiple myeloma
J774	Human macrophages
SEM	Scanning electron microscopy
XRD	X-ray diffraction
TGA	Thermogravimetric analysis
FITC	Annexin V-fluoresce isothiocyanate
LDH	Lactate dehydrogenase
HL-770	Normal liver cells
HepG2	Liver hepatocellular carcinoma
MTT	3-(4,5-dimethylthiazol-2-yl)-2,5-dipheny-ltetrazolium bromide
DAPI	4’,6-diamidino-2-phenylindole
PI	Propidium iodide
IC50	Half-maximal inhibitory concentrations
DRG	Rat neonatal organotypic dorsal root ganglion
DMEM	Dulbecco’s modified Eagle’s medium
DMSO	Dimethyl sulfoxide
FBS	Fetal bovine serum
